# Using the A/T/N Framework to Examine Driving in Preclinical Alzheimer’s Disease

**DOI:** 10.3390/geriatrics3020023

**Published:** 2018-05-02

**Authors:** Catherine M. Roe, Ganesh M. Babulal, Sarah H. Stout, David B. Carr, Monique M. Williams, Tammie L. S. Benzinger, Anne M. Fagan, David M. Holtzman, Beau M. Ances, John C. Morris

**Affiliations:** 1 Knight Alzheimer Disease Research Center, Department of Neurology, Washington University School of Medicine, St. Louis, MO 63110, USA; babulalg@wustl.edu (G.M.B.); shstout@wustl.edu (S.H.S.); bances@wustl.edu (B.M.A.); 2 Department of Medicine, Washington University School of Medicine, St. Louis, MO 63110, USA; dcarr@wustl.edu; 3 VITAS Healthcare, St. Louis, MO 63110, USA; Monique.Williams@vitas.com; 4 Knight Alzheimer Disease Research Center, Departments of Radiology and Neurosurgery, Washington University School of Medicine, St. Louis, MO 63110, USA; benzingert@wustl.edu; 5 Knight Alzheimer Disease Research Center, Department of Neurology, the Hope Center for Neurological Disorders, Washington University School of Medicine, St. Louis, MO 63110, USA; fagana@wustl.edu (A.M.F.); holtzman@wustl.edu (D.M.H.); 6 Knight Alzheimer Disease Research Center, Departments of Neurology, Neurosurgery, Occupational Therapy, Pathology and Immunology, Physical Therapy, Washington University School of Medicine, St. Louis, MO 63110, USA; jcmorris@wustl.edu

**Keywords:** Alzheimer disease, preclinical Alzheimer disease, dementia, driving, biomarkers, cerebrospinal fluid, imaging

## Abstract

The A/T/N classification system is the foundation of the 2018 NIA-AA Research Framework and is intended to guide the Alzheimer disease (AD) research agenda for the next 5–10 years. Driving is a widespread functional activity that may be particularly useful in investigation of functional changes in pathological AD before onset of cognitive symptoms. We examined driving in preclinical AD using the A/T/N framework and found that the onset of driving difficulties is most associated with abnormality of both amyloid and tau pathology, rather than amyloid alone. These results have implications for participant selection into clinical trials and for the application time of interventions aimed at prolonging the time of safe driving among older adults with preclinical AD.

## 1. Introduction

By 2050, the United States will face a significant increase in the older adult population (age > 65 years), a higher prevalence of Alzheimer disease (AD) and a related increase in older drivers (1 in 4 drivers) [1,2]. While age-related changes (e.g., cataracts, reduced reaction time) is a risk factor for driving decline, AD is also an important risk factor for higher crash risk and resulting injury and mortality [3,4,5]. Preclinical AD is defined as the long asymptomatic stage without cognitive decline but presents with biomarker evidence indicating that the pathophysiological process has begun [6]. Prior work has shown that cognitively normal older adults with preclinical AD make more errors and are faster to receive a marginal or fail rating on a standardized road test compared to those without preclinical AD [7,8]. While past research has used a dichotomous classification of biomarker status (higher vs. lower) coupled with cognitive status, this study employed the A/T/N classification system to examine the relationship between AD biomarkers and driving performance as evaluated on a standardized road test [7,8].

The A/T/N classification system [9] is the foundation of the new 2018 NIA-AA Research Framework [10], which was developed to guide observational and interventional research in AD. Participants are assigned to one of eight A/T/N profiles based on whether amyloid (A), tau (T) and neurodegeneration (N) biomarkers are normal or abnormal. In turn, these eight profiles comprise four categories: AD (amyloid and tau both abnormal), AD pathophysiology (amyloid but not tau, abnormal) and normal (amyloid, tau and neurodegeneration normal) [10]. The framework is intended to guide the AD research agenda for the next 5–10 years [10].

Clinical trials testing potential treatments in the preclinical and early AD stages will be an important part of that agenda. These trials require evidence of both clinically meaningful cognitive and functional outcomes to demonstrate effectiveness.

Driving is a widespread functional activity that declines with symptomatic AD [8]. Recent work links driving and AD biomarker abnormalities among older adults who are cognitively normal [8,11]. Since the A/T/N framework is based solely on biomarkers, it may be particularly useful in investigation of functional changes in pathological AD before onset of cognitive symptoms. Thus, the purpose of this study is to examine driving in preclinical AD using the A/T/N framework.

## 2. Materials and Methods

Detailed information on study participants and methodology have been published [8]. Briefly, participants had normal cognition (Clinical Dementia Rating [CDR] [12] = 0), were aged 65 years and older, drove an automobile at least once per week and had amyloid imaging and/or cerebrospinal fluid (CSF) collection within 2 years prior to, or 6 months after, the baseline assessment. At baseline and subsequent annual visits participants took part in clinical assessments, self-reported their current driving practices using the Driving Habits Questionnaire (DHQ) [13] and completed an on-road driving test. The Washington University Road Test (WURT) is a 45-min, in-traffic, predetermined route progressing from a quiet parking lot to more complex traffic conditions as safety permits [14]. The participant drives a mid-size sedan (with dual brakes), while the driving evaluator in the front seat provides directions, maintains vehicle safety and evaluates driving performance. At the end of the WURT, the participant receives a global rating (pass, marginal, fail) via a standardized scoring system. A pass rating is defined as minimal to no errors/no safety concerns where the driver demonstrates competency in all aspects of the test and across the different levels of traffic densities. A marginal rating is defined as moderate to minimal errors and some safety concerns where behaviors like rolling stops, inconsistent scanning or inability to maintain appropriate speed are observed but infrequent. A fail rating is defined as high to moderate errors and significant safety concerns where the driver demonstrates behaviors like failing to yield to a vehicle/pedestrian or running traffic light, which may lead to a crash anytime during the test. Since all participants were cognitively normal, a rating of fail is extremely rare, while a marginal rating may reveal some problems. As a result, the overall rating was dichotomized into marginal/fail and pass in the main analyses.

*Ethical Statement.* Study protocols were approved by the Washington University Human Research Protection Office (#201706043) St. Louis, MO, USA and written informed consent was obtained. Investigations were carried out following the rules of the Declaration of Helsinki.

*Statistical analyses.* Each participant was assigned to one of the eight possible A/T/N profiles (A+/T+/N+, A+/T+/N−, A+/T−/N+, A+/T−/N−, A−/T+/N+, A−/T+/N−, A−/T−/N+, A−/T−/N−) [10]. Amyloid abnormality on PET imaging was operationalized as a florbetapir (F-AV-45) standardized uptake value ratio (SUVR) of ≥1.219 [11] or an SUVR ≥ 1.31 for Pittsburgh Compound B [8]. An “A+” designation was assigned when either amyloid imaging or CSF Aβ_42_ values (based on date and assay [8]) were abnormal. “T+” was assigned when CSF ptau_181_ ≥ 67 pg/mL and “N+” when tau ≥ 339 pg/mL [15]. Three A/T/N groups reflecting normal biomarkers, AD pathology, AD and suspected non-Alzheimer’s pathology [SNAP] were then constructed based on the A/T/N classifications [10]. Age-adjusted differences between the A/T/N groups at baseline on everyday self-reported driving practices (driving space, miles driven, number of places visited and number of trips over the previous year) [13] were examined using general linear models. Kaplan-Meier curves and Cox proportional hazards models adjusting for age, tested differences in the time from baseline to first rating of Marginal or Fail (vs. Pass) on the yearly driving test as a function of A/T/N group. Linear mixed models, adjusted for age, tested self-reported decline over time on everyday driving practices assessed using the DHQ. 

Given the current debate regarding whether SNAP is part of the AD pathological process, or reflects underlying tauopathy or other pathologies [16,17], we first analyzed the data excluding participants with SNAP, then reanalyzed the data including the SNAP group.

## 3. Results

A total of 157 participants with a mean ± SD age of 72.6 ± 4.9 years and education of 16.3 ± 2.6 years met inclusion criteria (Table 1). Seventy-three (46.5%) were normal (A−/T−/N−), 25 (15.9%) had AD pathology (A+), 25 (15.9%) had AD (A+/T+) and 34 (21.7%) had suspected non-Alzheimer’s pathophysiology (SNAP [10]). Participants with longitudinal data (N = 126) were followed up to 4.9 years (mean ± SD = 2.7 ± 1.0). Only two participants with marginal/fail ratings received a rating of fail (both within the first two years of follow-up), with the remaining participants who received marginal/fail ratings rated as marginal.

**Figure 1:**
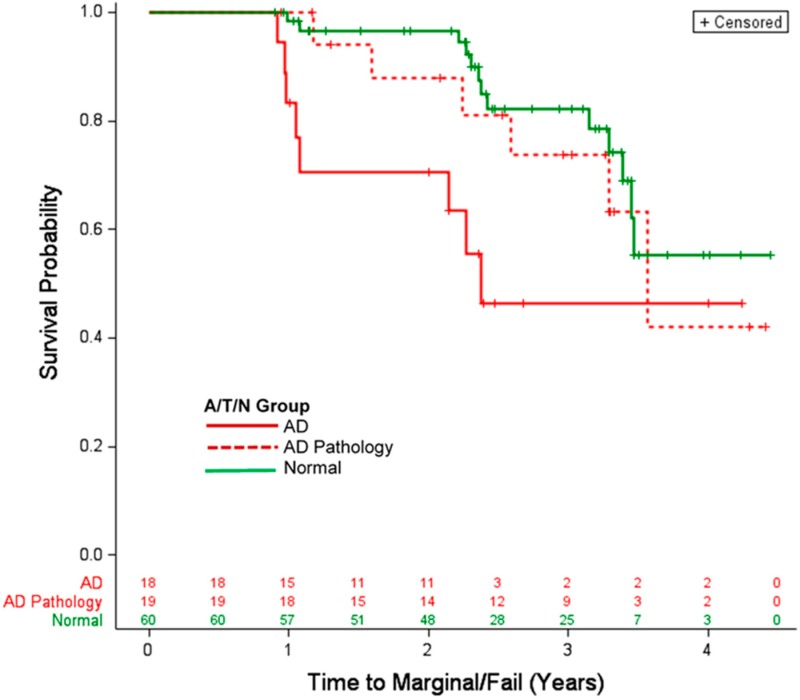
Time from baseline in years to receiving a Marginal or Fail rating as a function of A/T/N parent group membership. Numbers below the X-axis indicate the number of participants at risk at that time.

*Analyses without the SNAP group*. There were no differences between the A/T/N groups in everyday driving behaviors at baseline driving evaluation as reported on the DHQ (*p* > 0.212), nor in decline of DHQ-reported behaviors with time (*p* > 0.533). There were significant unadjusted (Log-rank test, *p* = 0.024; Wilcoxon, *p* = 0.001; Figure 1) and adjusted (*p* = 0.031) effects of A/T/N group on time from baseline to a marginal/fail rating on the driving test. Participants with AD received a marginal/fail rating earlier than those with normal A/T/N biomarkers (HR = 3.36, 95%CI = 1.37–8.25, *p* = 0.008), whereas there was no difference between those with normal A/T/N biomarkers and AD pathology (HR = 1.53, 95%CI = 0.59–4.02, *p* = 0.385) in time to a marginal/fail rating.

**Figure 2:**
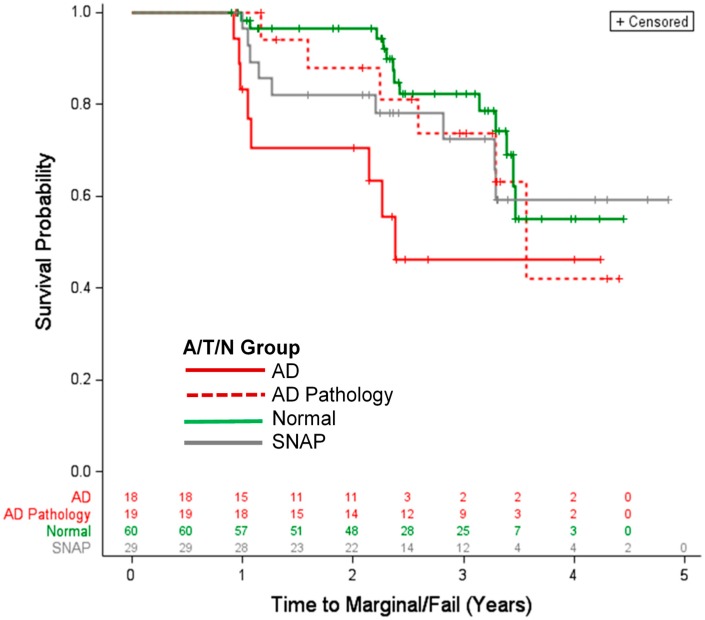
Time from baseline in years to receiving a Marginal or Fail rating as a function of A/T/N parent group membership, including participants with suspected non-Alzheimer’s pathophysiology (SNAP). Numbers below the X-axis indicate the number of participants at risk at that time.

*Analyses including the SNAP group*. As in the previous analyses, the A/T/N groups did not differ in everyday driving behaviors at baseline driving evaluation (*p* > 0.108), nor in decline of these behaviors with time (*p* > 0.184). With inclusion of the SNAP group, the unadjusted (Log-rank test, *p* = 0.054; Wilcoxon test = 0.004; Figure 2) and adjusted (*p* = 0.068) *p*-values reflecting differences between the A/T/N groups on time from baseline to a marginal/fail rating on the driving test increased. Participants with AD received a marginal/fail rating earlier than those with normal A/T/N biomarkers (HR = 3.36, 95%CI = 1.37–8.20, *p* = 0.008), whereas there was no difference between those with normal A/T/N biomarkers and AD pathology (HR = 1.50, 95%CI = 0.57–3.93, *p* = 0.410) or SNAP (HR = 1.42, 95%CI = 0.60–3.35, *p* = 0.429) in time to a marginal/fail rating.

## 4. Discussion

Examination of driving behavior using the A/T/N framework showed no associations between preclinical AD and cross-sectional, self-reported driving behavior at baseline, nor in changes in self-reported driving behavior with time. However, this framework predicted time to onset of objectively-measured driving problems on a standardized road test when only AD, AD pathology and normal groups were considered. Specifically, we found that the onset of driving difficulties is most associated with abnormality of both amyloid and tau pathology, rather than amyloid alone. These results suggest that in addition to cognition, the biomarker-based A/T/N framework may prove useful in guiding research that involves functional outcomes, such as driving. 

Twenty-two percent of our participants fit into the SNAP group at baseline, a figure similar to that shown in other research samples of cognitively normal individuals [16,17]. There is uncertainty and controversy regarding what the SNAP group represents, with some arguing that given the relatively high prevalence of SNAP among cognitively normal older adults, it should be considered as part of the AD pathological spectrum and that the amyloid-centric view of AD is flawed. Others believe that SNAP may reflect distinct pathologies separate from AD, such as cerebrovascular disease, hippocampal sclerosis, or Parkinson’s disease [16,17]. For this reason, we analyzed the data both with and without the SNAP group. 

Upon reanalysis of the data including the SNAP group, the age-adjusted relationship between A/T/N and onset of driving problems was no longer significant. This is likely due to the relatively larger number of SNAP participants compared to the AD and AD pathology groups and the position of the SNAP survival curve relative to the others. As shown in Figure 2, the onset of driving difficulties among the SNAP groups driving behavior falls somewhere between that of the normal and AD groups. Using the onset of cognitive problems as an outcome, others have reported that the SNAP group tends to develop cognitive symptoms earlier than the persons in the normal group but later than those with biological AD [16,17].

As noted, most persons in our study who showed difficulty on the driving test received a “marginal” rating and the membership in the AD group should not be used as an indication that an individual should stop driving. Driving cessation is associated with several negative consequences, including higher rates of admission to long-term care facilities, higher prevalence of depression, faster decline in overall health and higher overall rates of mortality [18].

There are some limitations to this study. Importantly, the sample size of some of the A/T/N groups were small, with less than 20 participants with longitudinal data in each of the AD and AD pathology groups and therefore these results should be considered as preliminary. We will continue to follow this cohort to examine associations between the A/T/N framework and driving behavior among more people over a longer follow-up period. As with other standardized road tests [19,20], the WURT is a controlled condition with limited inference to actual daily driving behaviors and may be influenced by factors such as anxiety and rater subjectivity. Participants were predominately Caucasian, well-educated and willing to complete, biomarker testing for cerebrospinal fluid and amyloid imaging, annual clinical and neuropsychological testing and therefore they may not be representative of the general population. Since, the A/T/N framework is a newer AD classification system, further work is needed to confirm these findings on the relationship between driving and preclinical AD in larger and more diverse groups.

## 5. Conclusions

If replicated, these results may have implications for participant selection into clinical trials and for the application time of interventions aimed at prolonging the time of safe driving among older adults with preclinical AD. Because the onset of driving difficulties may be most associated with abnormality of both amyloid and tau pathology, rather than amyloid alone, our results suggest that the best time to introduce interventions may be once amyloid biomarkers are abnormal but before abnormalities are present in both tau and neurodegeneration. 

## Figures and Tables

**Table 1:** Baseline demographic, biomarker and psychometric test performance for each group.

	AD (N = 25)		AD Pathology (N = 25)		Normal (N = 73)		SNAP (N = 34)		Total (N = 157)
	Mean/N	SD/%	Mean/N	SD/%	Mean/N	SD/%	Mean/N	SD/%	*p*-Value
Demographics									
Age, y	73.9	5.2	72.5	5.2	71.8	4.0	73.5	5.8	0.159
Education, y	16.0	2.5	16.4	2.8	16.6	2.6	16.0	2.5	0.612
Women, N	10	40.0%	14	56.0%	35	48.0%	20	58.8%	0.469
Race, N									0.940
AA	2	8.0%	3	12.0%	10	13.7%	4	11.8%	
Caucasian	23	92.0%	22	88.0%	62	84.9%	30	88.2%	
More than one race	0	0.0%	0	0.0%	1	1.4%	0	0.0%	
APOE4	13	52.0%	12	48.0%	13	17.8%	34	17.7%	<0.001
Biomarker values									
Amyloid positive, N									
Adjusted Aβ_42_	−1.1	0.4	−1.0	0.5	0.4	0.8	0.7	0.8	<0.001
tau, pg/mL	691.2	235.5	242.1	69.4	234.5	55.1	432.3	97.4	<0.001
ptau_181_, pg/mL	111.2	30.7	43.7	11.1	46.3	9.8	77.5	20.2	<0.001
Psychometric performance									
SRT Free Recall	29.7	8.2	32.3	4.8	32.4	5.6	31.9	5.5	0.249
Trailmaking A	30.9	10.7	29.2	9.5	28.9	7.9	29.8	7.8	0.776
Trailmaking B	83.0	33.3	75.7	29.3	76.1	30.5	79.5	30.8	0.805
Animal Naming	20.3	6.9	20.3	4.9	22.0	6.0	21.4	5.1	0.630

Abbreviations: AD = Alzheimer’s disease; SNAP = Suspected non-Alzheimer Disease Pathophysiology; SD = standard deviation; AA = African American; SRT = Selective Reminding Test.
